# Juvenile Metachromatic Leukodystrophy in a Seven-Year-Old Child With a Familial History: A Case Report Suggesting Saposin B Deficiency

**DOI:** 10.7759/cureus.102687

**Published:** 2026-01-31

**Authors:** Abdisalam O Hassan, Raja Arrab, Youssef Benchchehab, Insaff AL Ammari, Nezha Dini

**Affiliations:** 1 Pediatrics, Mohammed VI International University Hospital, Mohammed VI University of Health Sciences, Casablanca, MAR; 2 Pediatrics, Cheikh Khalifa International University Hospital, Mohammed VI University of Health Sciences, Casablanca, MAR

**Keywords:** demyelinisation, genetic diagnosis, juvenile-onset, metachromatic leukodystrophy, psap gene, saposin b deficiency

## Abstract

Metachromatic leukodystrophy (MLD) is a rare inherited disorder of the white matter with higher incidences in consanguineous populations. In children, manifestations vary with age of onset: early forms present with motor regression and developmental delay, whereas later forms begin with motor difficulties followed by behavioural or cognitive decline. We describe a 7-year-old boy with previously normal development who exhibited progressive motor deficits, speech difficulties, cognitive impairment, and loss of bladder control. Neurological examination revealed generalized hypotonia, areflexia, gait ataxia, and axial spasticity. Audiovisual function was preserved. Laboratory workup showed elevated urinary sulfatide levels despite normal enzymatic activity of the main sulfatide-degrading enzyme. Additional metabolic tests were unremarkable aside from signs of increased ketone bodies. Neuroimaging revealed white matter abnormalities consistent with a leukodystrophic process, and electrophysiological studies confirmed peripheral demyelination. Genetic analysis revealed a homozygous PSAP c.777G>A variant, affecting a gene essential for sulfatide degradation and suggesting a possible atypical form of MLD. This case highlights the diagnostic complexity of non-classical presentations and underscores the value of comprehensive metabolic and genetic evaluation. This case illustrates that MLD can present with normal enzymatic assays and highlights the importance of combined biochemical, neuroimaging, and genomic testing in children with rapid motor and cognitive decline.

## Introduction

Metachromatic leukodystrophy (MLD) is part of a heterogeneous group of inherited degenerative encephalopathies with autosomal recessive inheritance. It is characterized by the accumulation of sulfatide glycopeptides in lysosomes, leading to progressive demyelination of the central and peripheral nervous systems [1]. It belongs to a group of inherited white matter disorders. After excluding vascular and inflammatory causes, metabolic leukodystrophies should be considered in the differential diagnosis when unexplained white matter abnormalities are present. Genetic cerebral metabolic disorders are typically identified through metabolite quantification and/or the discovery of a pathogenic gene mutation. MLD typically presents in childhood with a wide range of symptoms, from early learning and behavioral disorders to gait and balance disturbances. The diagnosis is based on elevated urinary sulfatides, reduced or absent arylsulfatase A activity, and characteristic white matter signal abnormalities on brain MRI [2]. Treatment of MLD includes both currently available therapies and experimental approaches, such as enzyme replacement therapy. In the absence of treatment, the morbidity and mortality remain high. We report the case of a seven-year-old child admitted for gait and behavioral disorders, in whom a diagnosis of leukodystrophy was established.

## Case presentation

A 7.5-year-old male presented in January 2025 with progressive weakness of all four limbs, accompanied by balance and gait disturbances. He was born to first-degree consanguineous parents, and his immunizations were up to date according to the national schedule. Psychomotor development had been normal until 83 months of age. Six months prior to presentation, he began losing previously acquired motor abilities. This regression was marked by cognitive decline and behavioral disturbances, followed by the onset of gait impairment and muscular weakness. He required assistance to walk, was unable to sit without support, had speech difficulties, and developed sphincter incontinence, all of which significantly impacted his school performance. A similar case in the family was documented. On clinical examination, the child was alert but demonstrated disturbances in attention, with intermittent episodes of unresponsiveness. Neurological assessment revealed generalized hypotonia associated with visible muscle atrophy. A sensorimotor deficit was noted in all four limbs, affecting both superficial and deep sensitivity. Osteotendinous reflexes were absent throughout. Gait assessment showed a broad-based, unstable pattern with a star-shaped walking trajectory and the presence of equinovarus foot deformity.

Muscle tone evaluation revealed mild spasticity in the upper trunk, graded 1 on the Modified Ashworth Scale. Cranial nerve examination was unremarkable, with preserved visual and auditory functions. In biological analyses, the complete blood count showed leukocytosis (17,460/mm³) with neutrophil predominance, hemoglobin at 13 g/dL, and platelet count of 402,000/mm³. C-reactive protein (CRP) and procalcitonin (PCT) levels were negative. In the biochemical Profile, plasma homocysteine was 10.78 µmol/L. Elevated concentrations of sulfatides were noted, specifically C16:0, C16:0-OH, and C16:1-OH. Table 1 summarizes the patient’s biochemical findings, including elevated sulfatides, normal arylsulfatase A activity and very long chain fatty acids.

**Table 1:** Biochemical profile of the patient compared to reference values

Parameter	Patient Result	Normal Reference Value
Homocysteine [µmol/L]	10.78	< 15
C16:0 [µmol/L]	2.90	< 1.34
C16:0-OH [µmol/L]	8.24	< 0.30
C16:1-OH [µmol/L]	0.78	< 0.11
Arylsulfatase A [nmol/punch/h]	1.26	> 0.08
Phytanic Acid [µmol/L]	0.68	< 4.70
Pristanic Acid [µmol/L]	< 0.30	< 0.55

Organic acids were marked by elevation of ketone bodies with mild dicarboxylic aciduria (trace amounts) without evidence of associated lactic acidosis. This global hyperaminoacidemic profile is detailed in Table 2, which shows elevated concentrations of several amino acids compared to reference values.

**Table 2:** Amino acid chromatography results

Amino Acid	Patient Result	Reference Range
Leucine [µmol/L]	170	110-158
Isoleucine [µmol/L]	92	57-77
Valine [µmol/L]	313	216-290
Phenylalanine [µmol/L]	81	48-68
Tyrosine [µmol/L]	76	48-84
Tryptophan [µmol/L]	65	30-70
Methionine [µmol/L]	48	20-32
Threonine [µmol/L]	216	86-146
Serine [µmol/L]	214	111-163
Glutamic Acid [µmol/L]	184	111-163
Aspartic Acid [µmol/L]	205	130-210
Lysine [µmol/L]	266	147-225
Arginine [µmol/L]	86	54-104
Histidine [µmol/L]	87	73-97
Glycine [µmol/L]	407	179-269
Alanine [µmol/L]	211	214-336
Proline [µmol/L]	175	150-180
Cystine [µmol/L]	31	70-98
Ornithine [µmol/L]	90	42-90
Citrulline [µmol/L]	20	18-34

**Figure 1:**
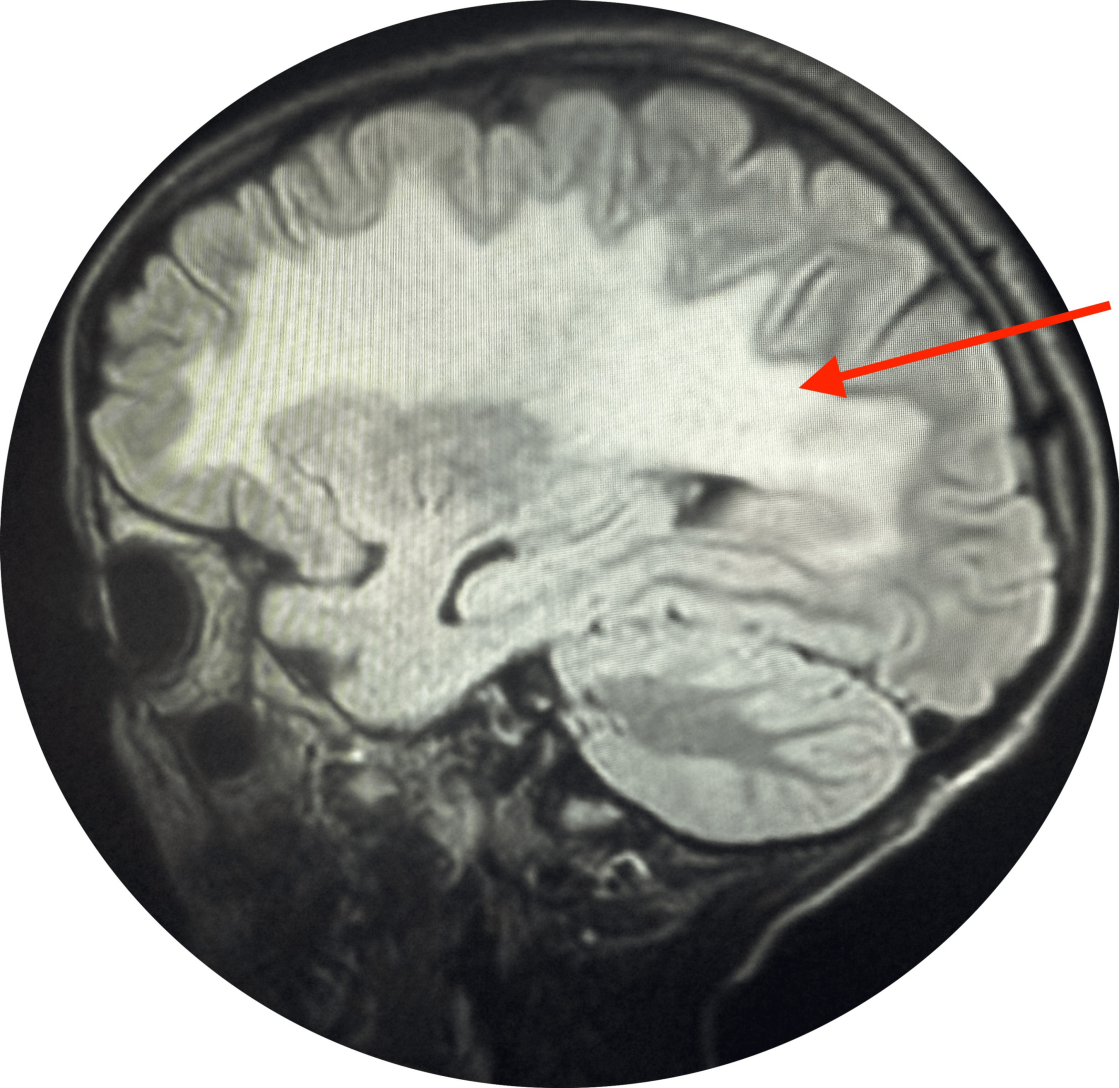
MRI sagittal plane

**Figure 2:**
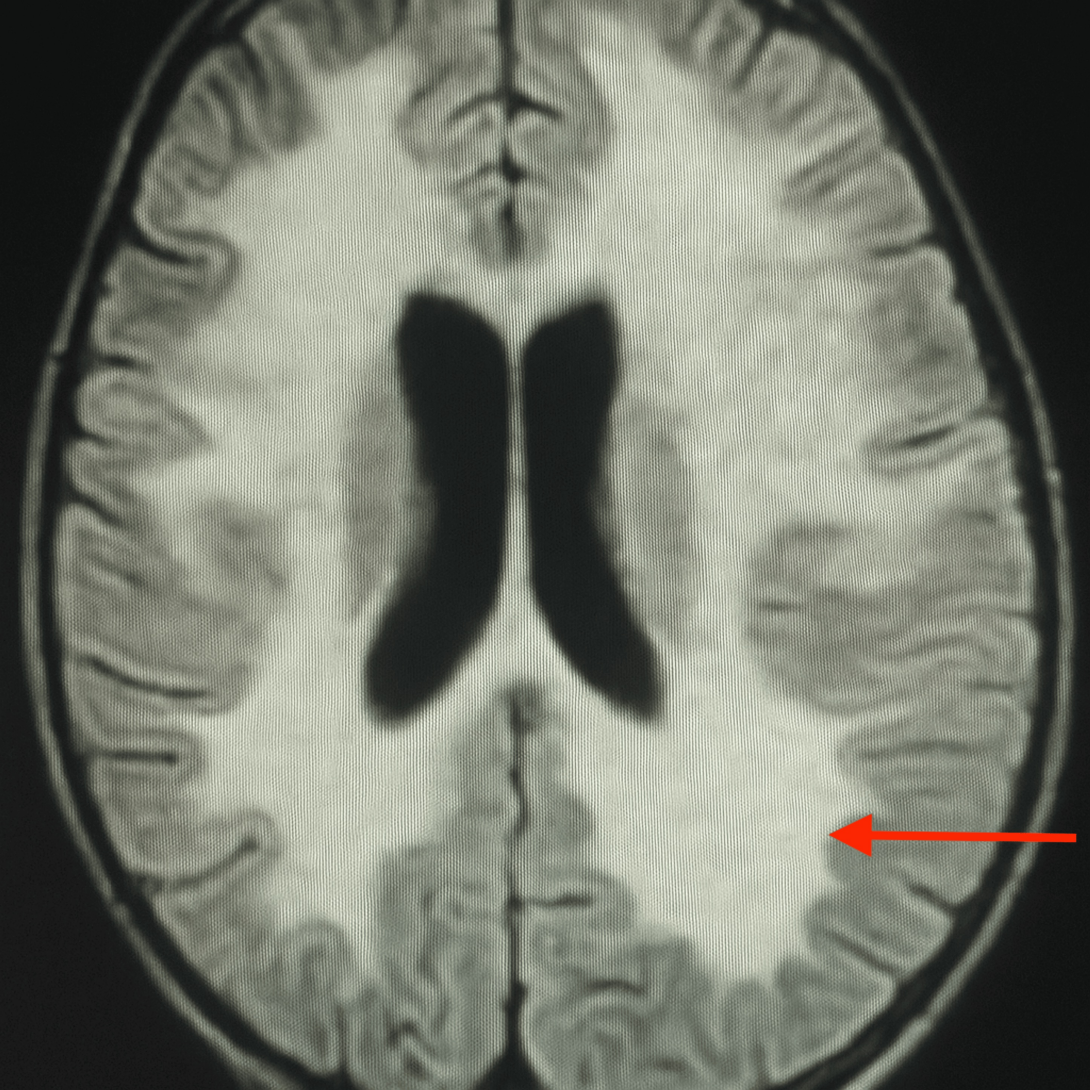
MRI axial

On imaging and neurophysiology, the chest radiograph was normal. Brain MRI showed hyperintense signal abnormalities in the subcortical and periventricular white matter, as well as in the centrum semiovale on FLAIR and diffusion sequences with elevated ADC values, producing a «butterfly-wing» appearance suggestive of MLD as revealed in Figures 1-2. Spinal MRI was normal.

Electroencephalogram (EEG) demonstrated a normal background rhythm without abnormal waveforms. Nerve conduction studies (ENMG) revealed features consistent with a demyelinating polyneuropathy affecting all four limbs. Sensory nerve conduction studies could not be performed due to a lack of cooperation. Whole-exome sequencing identified a homozygous missense variant in the PSAP gene (c.777G>A; p.Met259Ile), not previously reported in population databases (gnomAD v4.1.0). Classified as a variant of uncertain significance (VUS), it is predicted to affect splicing and is associated with MLD due to SAP-B deficiency (OMIM: 249900). Table 3 summarizes the clinical, molecular, and computational findings. Parental testing and functional validation were recommended for variant reclassification. For therapeutic intervention, physiotherapy and psychomotor rehabilitation aimed at improving postural tone in standing and sitting positions, as well as oculo-manual and oculo-pedal coordination, were initiated. The therapeutic program also emphasized progressive muscle strengthening, balance retraining, and coordination exercises adapted to the child’s functional level.

**Table 3:** Exome sequencing findings EDTA: Ethylenediaminetetraacetic acid

Category	Details
Patient	Seven-years-old male
Sample Type	EDTA blood
Collection / Accession Dates	Collected: 2025-02-10; Accessioned: 2025-03-11
Symptoms	Leukodystrophy
Gene	PSAP
Genomic Position	10:71825837-C-T (GRCh38)
cDNA	NM_002778.4:c.777G>A
Protein	NP_002769.1:p.Met259Ile
Zygosity	Homozygous
Inheritance	Unknown
Variant Type	Missense
Population Data	Variant not observed in gnomAD: v4.10 dataset,
Computational & Functional Data	In silico tool predictions suggest a damaging effect of the variant on the gene or gene product [(3Cnet: 0.81(>0.75) sensitivity 0.96 and precision 0.92)]. In silico tools predict the variant after splicing and produce an abnormal transcript [SpliceAI: 0.85(>=0.2 moderate evidence for splicegenicity)]
Previously Reported Variant Data	Different missense changes at the same codon have been reported as of uncertain significance (ClinVar ID: VCV001909907)
Disease Association	Metachromatic leukodystrophy due to SAP-B deficiency (OMIM: 249900)
Validation	Not performed (high-quality variant)
Classification	VUS – Variant of Uncertain Significance
Interpretation	Homozygous PSAP variant of uncertain significance. Functional testing and parental analysis are recommended for reclassification.

## Discussion

There are currently more than 30 diseases classified as leukodystrophies, as identified by the Canadian Paediatric Surveillance Program. These include MLD, X-linked adrenoleukodystrophy, and Krabbe disease [3]. MLD is typically suspected in the presence of motor disturbances (e.g., gait abnormalities) and behavioral changes, often preceded or accompanied by cognitive decline in a child with previously normal psychomotor development [4].

MLD is classically divided into three clinical forms based on age at onset: The late-infantile form, the most common, begins before the age of two and progresses rapidly with motor decline, loss of acquired skills, and poor prognosis. The juvenile form, appearing between ages six and 10, has a slower course with motor difficulties, cognitive decline, and behavioral symptoms. The adult form, generally after age 10, progresses slowly and is dominated by cognitive and psychiatric symptoms, sometimes accompanied by motor impairment later in the disease course [5]. Our patient presented the juvenile form, combining progressive neuromotor deficits, cognitive regression, and behavioral disturbances.

Metabolic investigations typically supporting the diagnosis of MLD include elevated urinary sulfatides and/or reduced or absent arylsulfatase A (ARSA) enzymatic activity. In our patient, urinary sulfatides were elevated, while ARSA activity remained within normal limits. Although ARSA deficiency is the most common cause of MLD, normal enzymatic activity does not exclude the diagnosis. In such cases-particularly when clinical, biochemical, and radiological features are suggestive, molecular analysis of the PSAP gene is warranted. This testing may reveal mutations in the PSAP gene. PSAP encodes saposin B, a crucial activator protein required for sulfatide degradation. Thus, the presence of a homozygous PSAP variant in the context of normal ARSA activity strongly supports a diagnosis of saposin B deficiency-related MLD. Whole-exome sequencing (WES) has been shown to detect up to 42% of previously undiagnosed or genetically uncharacterized leukodystrophies and leukoencephalopathies [6]. In our case, WES identified a homozygous variant of uncertain significance in the PSAP gene, suggesting saposin B deficiency. We speculate that residual ARSA enzymatic activity, commonly observed in the juvenile form, may explain the patient's biochemical profile.

As the name suggests, leukodystrophy involves abnormalities of the central nervous system white matter. Therefore, any white matter abnormality observed on MRI should prompt evaluation for MLD after excluding inflammatory or vascular causes. MRI remains the most valuable imaging tool, regardless of the patient's age. In our case, white matter abnormalities were observed in the subcortical and periventricular regions [4].

Therapeutic options include hematopoietic stem cell transplantation, which involves ex vivo insertion of a functional copy of the ARSA gene into the patient's hematopoietic stem cells, followed by reinfusion. In 2020, the European Medicines Agency approved atidarsagene autotemcel (Libmeldy/Lenmeldy), an ex vivo autologous hematopoietic stem‑cell gene therapy, for pre‑symptomatic late‑infantile or early‑juvenile MLD and early‑juvenile patients who can still walk independently [7]. Long‑term follow‑up suggests sustained benefits. One limitation is that treatment becomes ineffective once clinical symptoms appear. Careful observation of early developmental delays in presymptomatic individuals could enable earlier diagnosis and significantly improve outcomes [8]. To achieve favorable clinical results, it is crucial to diagnose the disease at an early stage of progression. The fact that our patient has a cousin with the same disease further reinforces the need to include these disorders in neonatal screening programs [8,9]. Finally, the prognosis of our patient appears less severe than that of patients with the late-infantile form, which progresses more rapidly and is associated with shorter survival [9].

## Conclusions

MLD is a neurodevelopmental disorder characterized by progressive loss of previously acquired psychomotor skills, sensorimotor deficits, and demyelinating polyneuropathy. Diagnosis is based on enzymatic assays and/or identification of pathogenic genetic variants; in the absence of a known biochemical marker, diagnosis may rely on characteristic clinical and radiological findings. Brain MRI, the most informative imaging modality across all age groups, plays a key role in early detection by revealing subtle or even subclinical white matter lesions. Early initiation of multidisciplinary rehabilitation is essential to preserve functional abilities and slow disease progression. Moreover, longitudinal studies are warranted to assess long-term outcomes in individuals treated presymptomatically, and to elucidate genotype-phenotype correlations that may influence disease trajectory and therapeutic response.
